# Differential expression of plasma proteins and pathway enrichments in pediatric diabetic ketoacidosis

**DOI:** 10.1186/s10020-024-01056-7

**Published:** 2025-01-07

**Authors:** Paolo Spagnolo, Enis Cela, Maitray A. Patel, David Tweddell, Mark Daley, Cheril Clarson, Saverio Stranges, Gediminas Cepinskas, Douglas D. Fraser

**Affiliations:** 1 Medicine, Campus Bio-Medico University of Rome, Via Alvaro del Portillo 21, Rome, Italy 00128; 2 Physiology and Pharmacology, Western University, London, ON Canada N6A 3K7; 3 Epidemiology and Biostatistics, Western University, London, ON Canada N6A 3K7; 4 Computer Science, Western University, London, ON Canada N6A 3K7; 5 Pediatrics, Western University, London, ON Canada N6A 3K7; 6 Children’s Health Research Institute, London, ON Canada N6C 4V3; 7 Medicine, Western University, London, ON Canada N6A 3K7; 8 Family Medicine, Western University, London, ON Canada N6A 3K7; 9 Clinical Medicine and Surgery, University of Naples Federico II, Naples, Italy 80131; 10 Medical Biophysics, Western University, London, ON Canada N6A 3K7; 11 London Health Sciences Centre Research Institute (LHSC-RI), London, ON Canada N6A 5W9; 12 Clinical Neurological Sciences, Western University, London, ON Canada N6A 3K7; 13 Room A5-132, Victoria Research Laboratories, LHSC-VC, 800 Commissioners Road E., London, ON Canada N6A 5W9; 14 Anatomy and Cell Biology, Western University, London, ON Canada N6A 3K7; 15 Vector Institute for Artificial Intelligence, Toronto, ON Canada M5G 0C6

**Keywords:** Pediatric, Diabetic ketoacidosis, Proteomics, Protein expression, Pathways

## Abstract

**Background:**

In children with type 1 diabetes (T1D), diabetic ketoacidosis (DKA) triggers a significant inflammatory response; however, the specific effector proteins and signaling pathways involved remain largely unexplored. This pediatric case–control study utilized plasma proteomics to explore protein alterations associated with severe DKA and to identify signaling pathways that associate with clinical variables.

**Methods:**

We conducted a proteome analysis of plasma samples from 17 matched pairs of pediatric patients with T1D; one cohort with severe DKA and another with insulin-controlled diabetes. Proximity extension assays were used to quantify 3072 plasma proteins. Data analysis was performed using multivariate statistics, machine learning, and bioinformatics.

**Results:**

This study identified 214 differentially expressed proteins (162 upregulated, 52 downregulated; adj P < 0.05 and a fold change > 2), reflecting cellular dysfunction and metabolic stress in severe DKA. We characterized protein expression across various organ systems and cell types, with notable alterations observed in white blood cells. Elevated inflammatory pathways suggest an enhanced inflammatory response, which may contribute to the complications of severe DKA. Additionally, upregulated pathways related to hormone signaling and nitrogen metabolism were identified, consistent with increased hormone release and associated metabolic processes, such as glycogenolysis and lipolysis. Changes in lipid and fatty acid metabolism were also observed, aligning with the lipolysis and ketosis characteristic of severe DKA. Finally, several signaling pathways were associated with clinical biochemical variables.

**Conclusions:**

Our findings highlight differentially expressed plasma proteins and enriched signaling pathways that were associated with clinical features, offering insights into the pathophysiology of severe DKA.

**Supplementary Information:**

The online version contains supplementary material available at 10.1186/s10020-024-01056-7.

## Introduction

Diabetic ketoacidosis (DKA) is a serious complication of Type 1 diabetes (T1D) in children, marked by hyperglycemia, ketonemia, and metabolic acidosis (Castellanos et al. 2020; Dhatariya et al. 2020). DKA arises from an imbalance between insulin and counterregulatory hormones, including glucagon, cortisol, catecholamines, and growth hormone (Glaser 2005). The loss of pancreatic beta cells leads to elevated hormone levels, stimulating lipolysis and the release of free fatty acids (Dhatariya et al. 2020; Svart et al. 2016). These fatty acids undergo beta-oxidation to produce ketone bodies, resulting in proton accumulation that overwhelms the bicarbonate buffering system and causes anion gap metabolic acidosis (Alois and Rizzolo 2017; Laffel 1999). Prompt recognition and treatment of DKA are essential to prevent severe complications, including coma and death (Mahmud et al. 2007).

In children, DKA is associated with a systemic inflammatory response that may impact patient outcomes (Aon et al. 2024). This response involves complex interactions, including neutrophil activation and elevated levels of pro-inflammatory proteins such as cytokines (Karavanaki et al. 2011), chemokines (Omatsu et al. 2014), matrix metalloproteinases (Woo et al. 2016a; Garro and Chodobski 2017), and serine proteases (Woo et al. 2016b). Variations in markers like IL-1 and MMP-9 have been suggested to mediate the severity of DKA complications, including vasogenic brain edema and stroke (Eisenhut 2018). Increased endothelial permeability and the transfer of inflammatory proteins across the blood–brain barrier may contribute to this risk (Fahey and Doyle 2019; Rochfort et al. 2016). Notably, leukocyte-derived MMP-8 and MMP-9 are elevated in blood from pediatric DKA patients and are potential candidates for blood brain barrier compromise (Garro and Chodobski 2017). Additionally, proteinase-3 (PR3, or PRTN3), which increases due to neutrophil degranulation in pediatric DKA, may degrade brain endothelial tight junctions, increasing the risk of cerebral edema (Woo et al. 2016b).

Proteomic techniques that evaluate thousands of circulating proteins offer a comprehensive view of disease and injury mechanisms in children (Patel et al. 2024; Miller et al. 2021), and may aid our understanding of DKA pathophysiology. This study aimed to identify plasma proteins unique to severe DKA patients compared to age- and sex-matched insulin-controlled participants. Specifically, we sought to: (1) quantify a wide range of plasma proteins in pediatric severe DKA patients and controls using proximity extension assays (PEA); (2) analyze differential protein expression and distribution; (3) identify enriched signaling pathways in DKA; and (4) correlate these pathway changes with demographic, clinical, and biochemical variables. Our study represents the first comprehensive evaluation of the DKA proteome.

## Methods

### Study design and participants

Patients with T1D were recruited at the Children’s Hospital, London Health Sciences Centre (London, Ontario, Canada), a regional tertiary care center. DKA is diagnosed based on hyperglycemia (blood glucose > 11 mmol/L), bicarbonate levels < 15 mmol/L, and ketonuria, and classified as mild (venous pH < 7.3), moderate (pH < 7.2), or severe (pH < 7.1). We approached all DKA patients that were admitted to the pediatric critical care unit (PCCU) over a 2-year period and enrolled only those that were classified as ‘severe’. Insulin-controlled patients were enrolled from a T1D outpatient clinic and were without DKA for at least 3 months. A convenient sample size was employed as accurate sample size calculations are not possible with large scale proteomic studies where effect size and variance are unknown.

### Blood collection and processing

Blood samples intended for both proteomic analyses and routine DKA laboratory testing were collected at PCCU admission prior to insulin administration. In all cases, normal saline was being administered. Certified nursing personnel drew samples into citrate-containing tubes (Vacutainers; BD Biosciences, Mississauga, Canada), and then centrifuged at 1500×*g* for 15 min at 4 °C (Brisson et al. 2012; Gillio-Meina et al. 2013). The upper plasma layer was aliquoted into 250 µl portions and immediately frozen at − 80 °C. Plasma samples remained frozen until use, with freeze–thaw cycles avoided.

### Proximity extension assay

Plasma samples were analyzed using the Proximity Extension Assay (PEA) (Olink Explore 3072, Boston, MA), following previously established protocols (Lundberg et al. 2011; Assarsson et al. 2014). The PEA process consists of three main steps: (1) antibody binding: antibody pairs, each tagged with unique DNA oligonucleotides, bound specifically to their target proteins in the plasma; (2) proximity-induced hybridization and extension: when the oligonucleotides were in close proximity, they hybridized and were extended by DNA polymerase; and (3) barcode amplification and sequencing: the extended DNA barcodes were amplified and analyzed using next-generation sequencing on the NovaSeq Platform (Illumina Inc., San Diego, CA). Results were reported as relative quantification on a log2 scale of normalized protein expression (NPX) values. We applied rigorous quality control measures for immunoassay performance, detection, and hemolysis, ensuring all patient and control samples were suitable for analysis.

### Metadata analysis and quality control

All patients had their metadata analyzed. For the insulin-controlled group, available metadata included age, sex, BMI, and HbA1c. The DKA group also had data on blood pH, PCO_2_, PO_2_, HCO_3_, lactate, glucose, BUN, white blood cells, neutrophils, lymphocytes, and admission GCS. Patient metadata was matched to the PEA data, which underwent quality control before statistical analysis. Only features passing internal quality controls were included, while those with a missing frequency of 0.75 or more were excluded due to common detection below the PEA’s threshold.

A multi-step quality control (QC) procedure was applied to the pre-normalized dataset. First, outliers were identified. Second, principal component analysis (PCA) was performed. Third, pairwise univariate association tests were conducted based on factor properties: chi-squared tests for two categorical factors, ANOVA for categorical versus continuous factors, Spearman correlation for two continuous factors, and Cox proportional hazards models with Wald tests for survival versus categorical factors. P-values were transformed to − log10(P) for heatmap visualization.

Further QC involved assessing 34 samples for outliers using statistical summaries: Sum of Euclidean distances, Kolmogorov–Smirnov test statistic, Mean Pearson correlation, and Hoeffding’s D statistic. A sample was considered an outlier if it failed two or more of these tests. Additionally, 34 samples were manually reviewed using various visualizations, including factor associations with principal components, dynamic PCA plots, correlation heatmaps, density plots, and MA plots.

### Natural language processing

Exploratory expression analysis was conducted to identify physiological areas of interest in DKA patients. Protein expression tissue specificity data were retrieved from the UniProt Knowledgebase via the UniProt REST API, providing curated information on mRNA and protein expression levels across various cells and tissues. This data was processed using Natural Language Processing (NLP) with the Stanza Python package, integrated with spaCy (Python v. 3.10.4; spaCy v. 3.3.1; spaCy-Stanza v. 1.0.2; negspaCy v. 1.0.3).

An NLP named-entity recognition pipeline was established using the MIMIC package for preprocessing, negation detection, and leveraging the pre-trained Stanza BioNLP13CG Biomedical model. Negation detection utilized the NegEx implementation in negspaCy, filtering out negative expression terms with a modified clinical term set. Although primarily trained on cancer genetics and PubMed abstracts, the BioNLP13CG model offered detailed entity classification, distinguishing between cell types, anatomical systems, organs, tissues, and multi-level tissues, which were then aggregated into an organ system classification.

Detected organ system and cell type entities were manually grouped into keyword-based categories, and the frequency of these categories relative to relevant proteins was analyzed to identify physiological expression patterns.

### Statistical analysis

Normalized data were employed for statistical hypothesis testing to identify proteins significantly differing between sample groups. Comparisons were conducted using empirical Bayes moderated t-tests implemented through the limma R package, with P-values adjusted for multiple testing to control the false discovery rate. In each comparison (e.g., group A vs. group B), a positive log2 (fold change) indicates up-regulation in group A relative to group B, while a negative log2 (fold change) indicates down-regulation. To ensure that hemoconcentration did not yield exaggerated measurements in the severe DKA cohort, we confirmed that a 3% shift in expression levels did not significantly alter the protein expression results (assuming 6–9% dehydration with 1/3 of this volume reduction occurring intravascularly).

### GO term ORA enrichment

Proteins with significant changes (adjusted p-value < 0.05 and fold change ≥ 2) in the "severe DKA vs insulin-controlled" patient comparison (n = 214) were mapped to Entrez gene identifiers (n = 211) and analyzed using Gene Ontology (GO) term over-representation analysis (ORA). GO terms, categorized into three domains—molecular function, biological process, and cellular component—were retrieved using the GO.db package (v3.19.1). Molecular function describes the activities of gene products at the molecular level, biological process pertains to biological programs involving multiple molecular activities, and cellular component indicates the locations where gene products operate. Enrichment analysis was conducted with the clusterProfiler package, using a minimum set size of 10 and a maximum of 500 proteins per GO term for over-representation consideration. GO term definitions can be found at https://www.ebi.ac.uk/QuickGO/.

### Pathway associations

The GO over-representation analysis identified 38 pathways that had an adjusted p-value < 0.05. These pathways were further analyzed for association with clinical observations, using a graph network approach. The pathways and clinical variables are disjoint sets and thus can be represented as nodes in a bipartite graph. The edges in the graph represent the degree of association between a pathway and a clinical variable. To determine a given pathway’s association with a given clinical variable, the given pathway’s set of implicated proteins obtained using GO ORA was used as an indicator. For each protein, we evaluated two features: the correlation of the protein value with the clinical variable (using Pearson’s correlation coefficient $\rho$), and the protein’s relative importance *R* in differential expression relative to a comparator group. Taking inspiration from the volcano plot, which presents impact (log2 fold change) and significance (−log10 (adjusted p-value)) in a two-dimensional plane, we can combine these two features into a single measure of the protein’s relative importance:

$$\text{R} = \sqrt{{\left({\text{log}}_{2}\left(\text{fold chan}{\text{ge}}\right)\right)}^{2}+{\left({\text{log}}_{10}\left(\text{adjusted p-value}\right)\right)}^{2}}$$

Then, for each protein the weight *w* for its association with a clinical variable is given by

$$w=\uprho R$$

When the Pearson’s $\rho$ was significant for a given protein/clinical variable pair (with a Pearson’s p-value < 0.05), we added an edge with weight *w* to the graph network, representing a connection between the given pathway (via the protein) and the clinical variable (i.e., the nodes in the graph). Comparisons that were not significant were not included. By summing the edge weights over pathway/clinical variable pairs, we obtained a representation of the strength of association between the pathway and the clinical variable.

## Results

### Patient demographic and clinical data

**Fig. 1:**
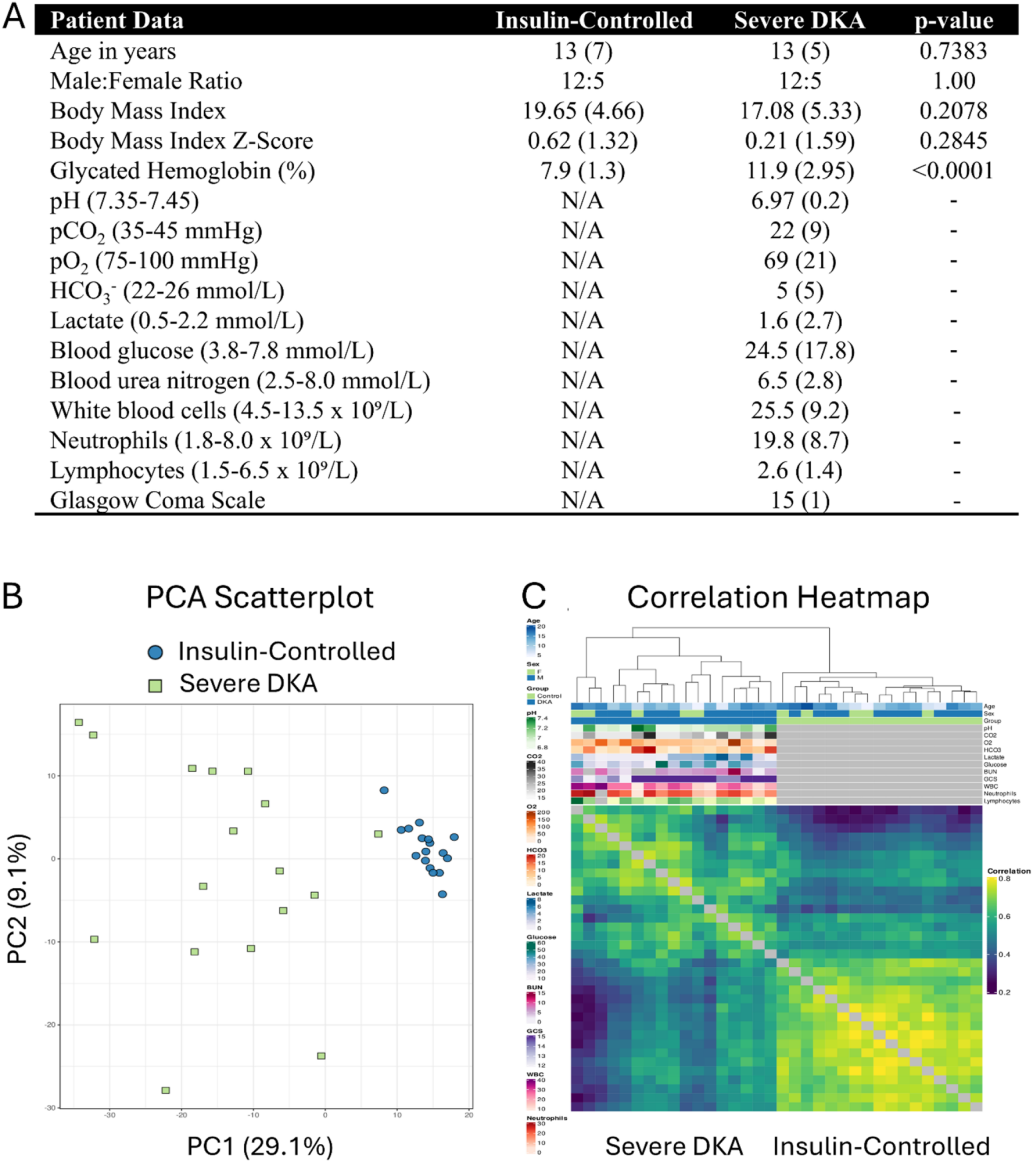
Patient variables and exploratory data analysis showing grouping and degree of correlation between samples. **A** Patient demographics and biochemistry, shown as median (IQR). **B** Scatterplots of the first two principal components from the normalized dataset. Colors represent the different cohorts in the dataset. Samples cluster according to one or more experimental factors to reveal the underlying cohorts. **C** Heatmap depicting pairwise Pearson correlations between samples of the normalized data. Samples are displayed along both the X and Y axes, with colour intensity indicating the degree of correlation (yellow: higher correlation, purple: lower correlation). Clustering, based on Euclidean distance, is illustrated by the dendrograms positioned above and to the left of the heatmap, with annotations provided for each sample

We examined the plasma proteomes of 17 individuals with severe DKA and 17 age- and sex-matched insulin-controlled participants. Their demographic, clinical and biochemical variables are listed in Fig. 1A. The Body mass index Z-scores were similar between groups; however, DKA patients had significantly higher HbA1C values than insulin-controlled participants (p < 0.0001), indicating elevated blood glucose over the previous 2–3 months. Based on blood gas tests, all DKA patients had high blood glucose and metabolic acidosis, along with leukocytosis due to neutrophilia. The GCS scores ranged from 12–15 (GCS 15, n = 10; GCS 14, n = 4; GCS 13, n = 1; GCS 12, n = 2). The duration of T1D ranged from 1–15 years for the insulin-controlled participants. Of the DKA patients, 7 were participants with known T1D (duration of T1D 2–11 years) and 10 participants were newly diagnosed T1D. None of the study participants had evidence of active infection or acute kidney injury (based on creatinine measurements within normal limits).

### Grouping and correlation analyses

Proteomic data was analyzed using PCA, a dimensionality reduction technique, to differentiate the two cohorts. Insulin-controlled patients clustered closely, while severe DKA patients exhibited greater heterogeneity and distinct separation from the insulin-controls (Fig. 1B). This separation highlights significant protein level differences between the groups, emphasizing the impact of severe DKA. A correlation heatmap further revealed stronger similarities within the insulin-controlled group compared to the severe DKA cohort (Fig. 1C).

### Differentially expressed proteins

**Fig. 2:**
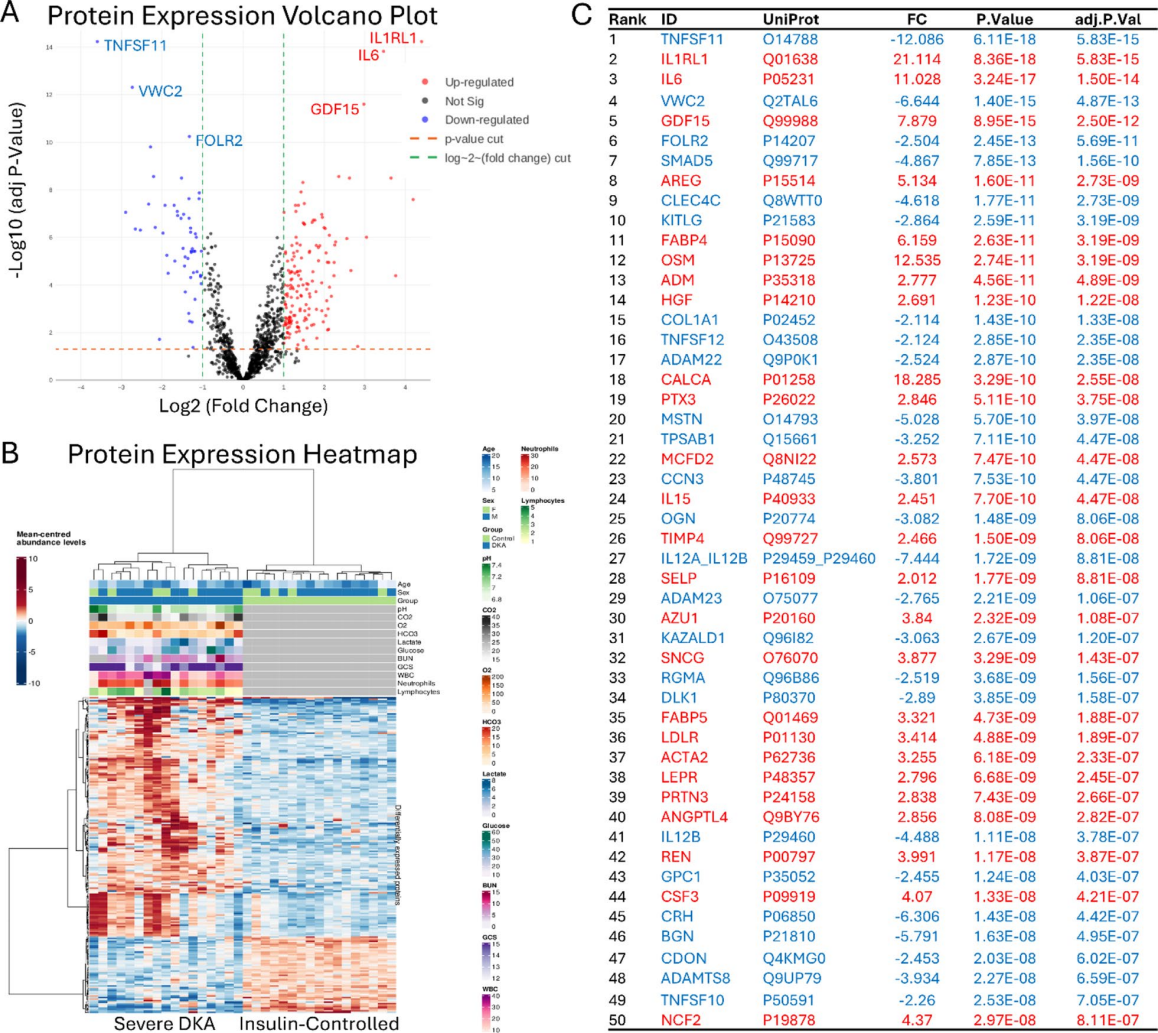
Association tests between severe DKA and insulin-controlled patients shown graphically and with a ranking of significant differentially expressed proteins. **A** Volcano plot depicting significance versus magnitude of protein expression changes. The x-axis represents the log2 fold change, while the y-axis shows −log10 transformed p-values. Proteins with significant differences between samples are colored: red for up-regulated and blue for down-regulated. For performance reasons, in cases with large datasets, the non-significant proteins (black) are shown as a representative sub-sample. Vertical green and horizontal orange lines denote the thresholds for fold change and p-value, respectively. **B** Heatmap illustrating protein intensity levels per sample, normalized to the average intensity across all samples. Proteins are listed along the Y-axis, and samples are along the X-axis. The colour scale indicates protein abundance: red for higher levels and blue for lower levels relative to the average. The heatmap displays up to 1000 proteins and 1000 samples, with features and samples selected randomly if the dataset exceeds these limits. **C** A list of the 50 leading differentially expressed proteins. Proteins upregulated in severe DKA patients are marked in red, while downregulated proteins are marked in blue. This list focuses on proteins exhibiting significant changes in abundance, based on adj P < 0.05 and a fold change > 2

Differentially expressed proteins (DEPs) were identified by comparing severe DKA patients to the insulin-controlled cohort, using a significance threshold of FDR-adjusted p-value < 0.05 and fold change (FC) ≥ 2. A total of 214 proteins were significantly different (Fig. 2A), with 162 upregulated and 52 downregulated in severe DKA. The heatmap in Fig. 2B shows protein intensity levels, with red and blue indicating higher and lower levels, respectively. The severe DKA cohort exhibited more proteins with increased expression compared to the insulin-controlled group. The top 50 significant proteins, including UniProt entries, fold changes, p-values, and adjusted p-values, are detailed in Fig. 2C. Notably, TNFSF11 and IL1RL1 showed the greatest changes, with TNFSF11 being less abundant and IL1RL1 more abundant in severe DKA samples. Other immune-related factors, such as CSF3, IL15, and IL12B, also exhibited significant differences between the groups. MMP8 and MMP9 were also significantly elevated in severe DKA, ranked 84/214 and 149/214, respectively (adj. P<0.0001).

### Organ and cell-type protein expression

**Fig. 3:**
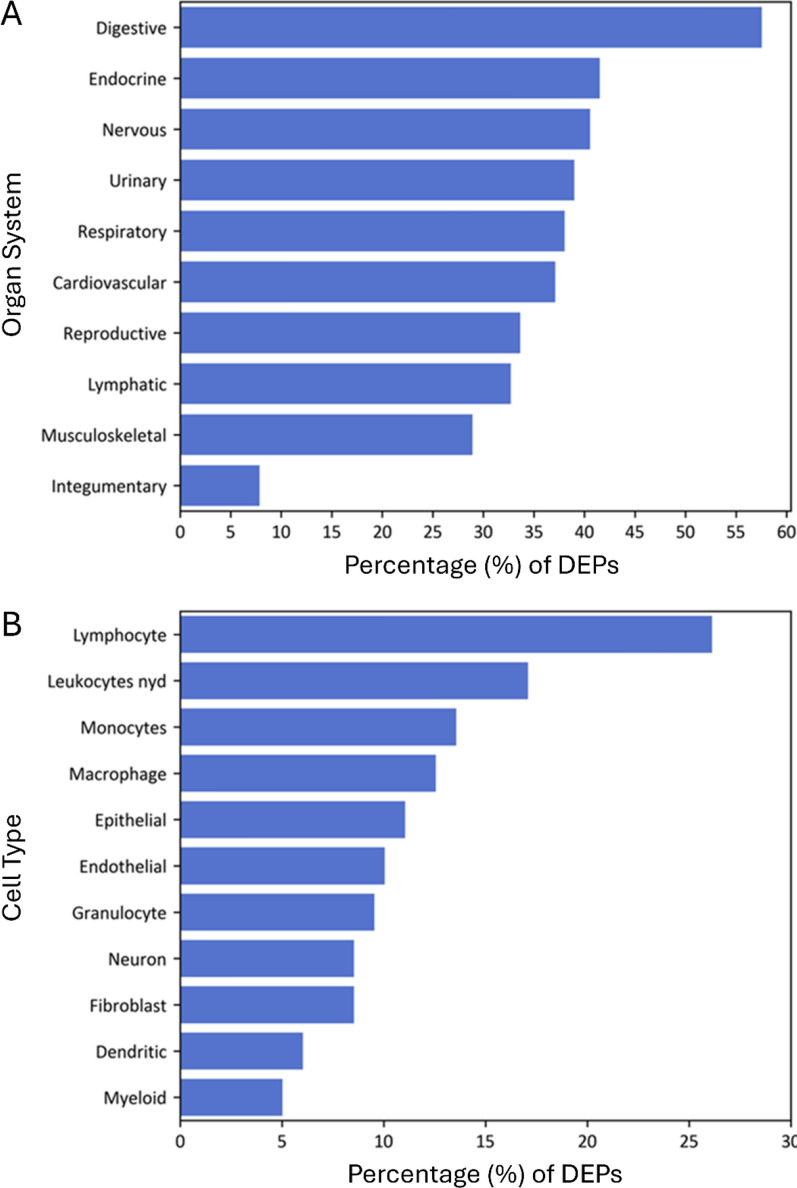
Frequency of differentially expressed proteins expression in major organs/body systems and cell types. **A** A bar plot demonstrates the percentage of differentially expressed proteins that are expressed in specific major organs and body systems as determined by Natural Language Processing (NLP). The organ system classification combines NLP-identified organs, tissue, multi-level tissue, and anatomical system entities. **B** A bar plot demonstrates the percentage of differentially expressed proteins that are expressed in specific cell-types as determined by NLP. Only 34% of proteins had UniProt cell expression information

The protein expression sites across various organ systems and cell types were examined in severe DKA patients using NLP. Data was available for 61% of DEPs, with the top three organ systems being digestive, endocrine, and neurological, based on UniProt expression data (Fig. 3A). For cell-type analysis, data was available for 34% of DEPs, which were primarily detected in WBCs, including lymphocytes, monocytes, and macrophages, as well as in epithelial and endothelial cells (Fig. 3B).

### Functional analysis

**Fig. 4:**
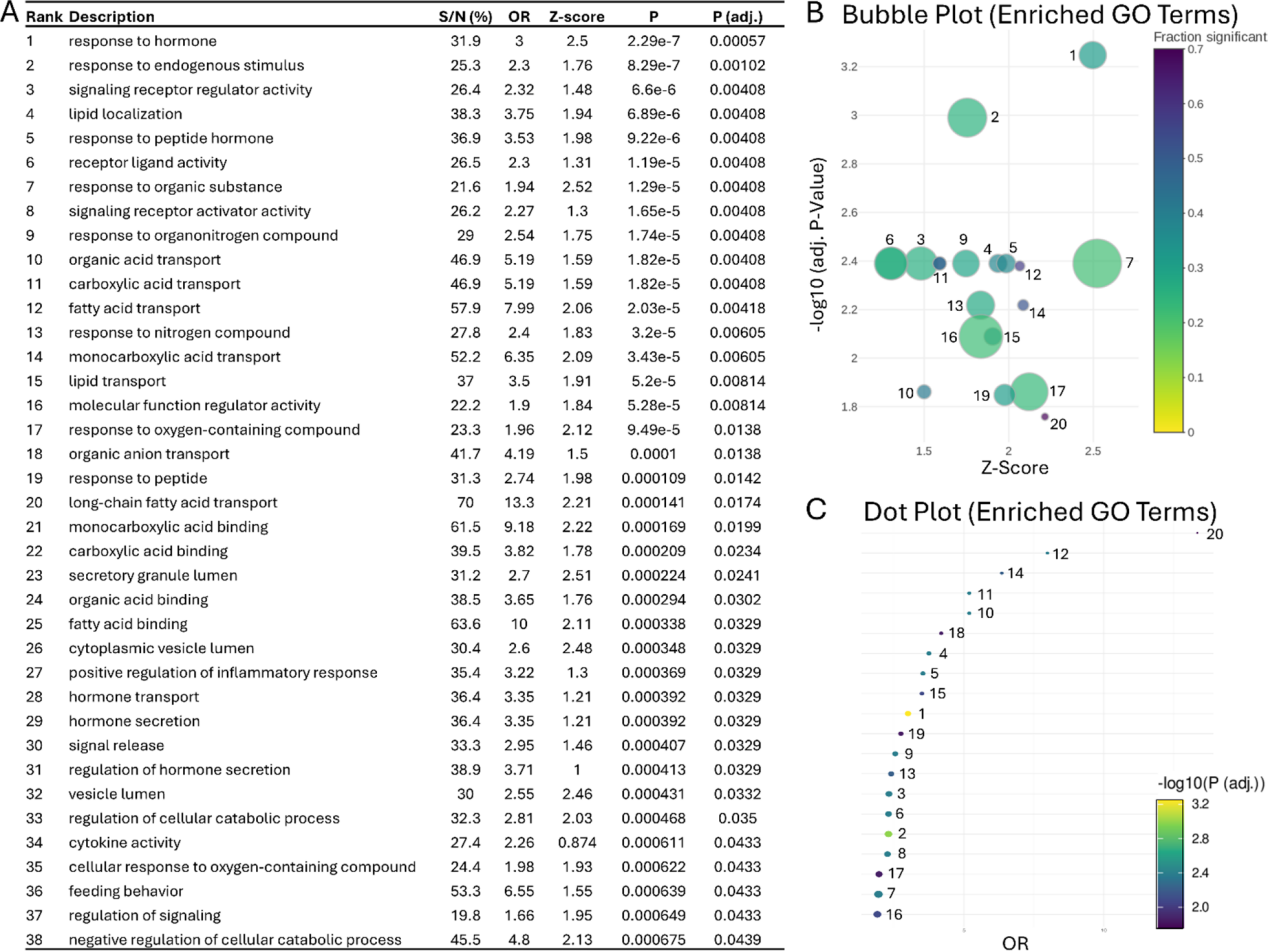
Functional analysis showing enriched pathways in Over-Representation Analysis (ORA) as a list and graphically. **A** A list that shows the pathways that are significantly enriched when comparing severe DKA to the insulin-controlled cohort. The pathways are ranked by their significance, with associated values, highlighting the differences in pathway activity linked to severe DKA. **B** Interactive bubble plot displaying significantly enriched Gene Ontology (GO) terms. The plot shows enrichment results with −log10(adjusted P-value) on the y-axis and enrichment Z-score on the x-axis, where Z = (Su − Sd) / √N. Here, N is the total number of proteins in the term, and Su and Sd represent the number of significant up-regulated and down-regulated proteins, respectively. Bubble size corresponds to term size, or the total number of proteins associated with the GO term, and colour indicates the fraction of significant features within each term. Only the top 20 GO terms based on adjusted P-value are shown. **C** Dot plot of significantly enriched GO terms with GO terms on the y-axis and odds ratio (OR) on the x-axis. The odds ratio is calculated as the ratio of observed significant proteins to the expected number by chance. Points are coloured based on −log10(adjusted P-value) and sized according to the number of proteins within each GO term. Only the top 20 terms based on adjusted P-value are displayed

Functional analysis using GO terms was performed to investigate the significant features identified in severe DKA. A significance threshold of an adjusted p-value < 0.05 was applied, revealing several enriched GO terms (Fig. 4A). The most significantly enriched pathway was "response to hormone", followed by other important pathways such as "positive regulation of inflammatory response", “lipoprotein assembly”, “lipid localization”, and “lipid transport”. A complete description of the statistically significant GO terms can be found in Supplemental Table 1. These enrichment pathways are visualized in Fig. 4B as a bubble plot, where the y-axis represents − log10(adjusted p-value), the x-axis shows the enrichment Z-score, the size of each bubble indicates the number of proteins associated with each GO term, and the color represents the fraction of significant features linked to each term. Figure 4C provides an alternative representation using a dot plot, where the x-axis shows the odds ratio (OR) for each GO term, and the y-axis lists the GO terms. In this plot, point size corresponds to the number of proteins within each GO term, while the color indicates − log10(adjusted p-value). Together, these analyses highlight key biological processes, including hormone response, inflammation regulation, and lipid metabolism, and the graphical plots offer a clear view of the statistical significance and associations of these pathways.

### Correlation of protein and clinical data

Correlation analysis of the top 50 differentially expressed proteins with clinical indicators is shown as a heatmap, after BH false discovery rate correction (Supplemental Fig. 1). IL1RL1 exhibited significant positive correlations with PO_2_ and WBC counts, and a strong negative correlation with GCS. Lactate (LEPR, ANGPTL4, CDON) and WBC counts (IL1RL1, CALCA, AZU1) had the most positive associations with various proteins.

### Correlation of enriched pathways and clinical data

**Fig. 5:**
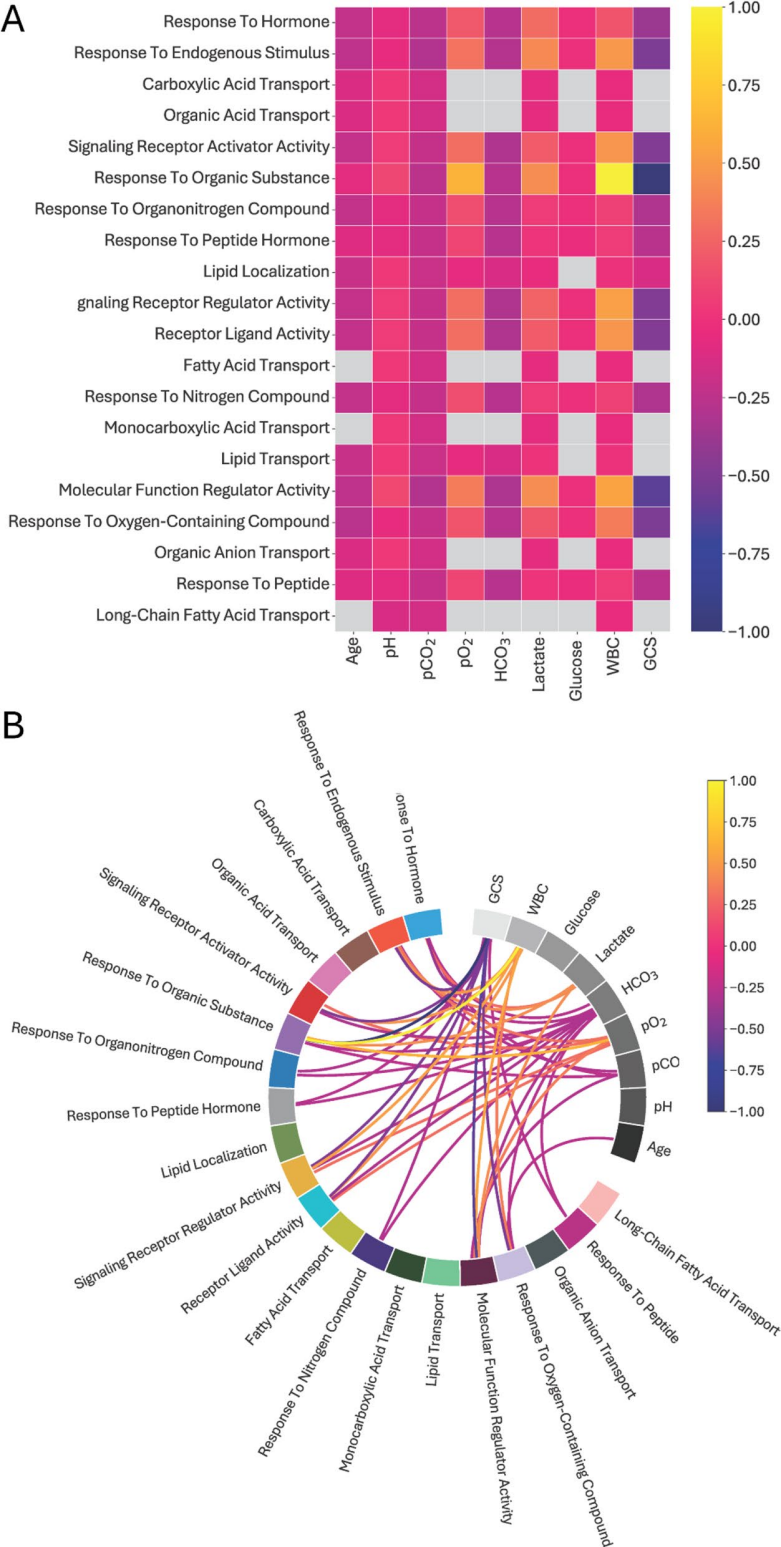
Heatmap and chord diagram displaying the associations between the top 20 enriched pathways prevalent in severe DKA patients identified through ORA analysis and clinical features. Clinical biochemistry and GCS were determined on admission to the pediatric critical care unit. **A** In the heatmap, the rows represent biological processes, while the columns the demographic features (gray color reflects non-significant association between pairs). Only comparisons with a Pearson's p-value less than 0.05 were considered significant and were included as edges in the graph; comparisons that were not significant were not included. **B** In the chord diagram, pathways are primarily positioned on the left, while clinical features are primarily positioned on the top right. Each edge represents the sum of the weights for implicated proteins in an enriched pathway, where the Pearson correlation coefficient $\uprho$ between the protein and a clinical feature is non-zero and significant (Pearson’s correlation p-value < 0.05). The edge colour indicates the direction of the correlation (dark blue for negative correlation, bright yellow for positive correlation), mapped to the range [− 1, 1] for convenience. Only associations < − 0.25 and > 0.25 (those with higher impact) are shown on the Chord diagram for greater clarity

The relationship between clinical features of severe DKA and enriched biological pathways, using pathway-associated proteins as indicators, are shown in Fig. 5. Associations are shown in both a heatmap and a chord plot. Most pathways exhibited negative correlations with pH and pCO_2_, particularly reflecting the metabolic and respiratory disturbances in DKA. In contrast, pathways related to molecular regulation and response, such as "Molecular Function Regulator Activity" and "Response to Endogenous Stimulus," showed positive correlations, suggesting increased activity in response to metabolic stress. A key finding was the positive correlation between WBC count and the "Response to Organic Substance" pathway, indicating immune activation during DKA. Lactate and PO_2_ were also positively correlated with metabolic and respiratory pathways. Conversely, GCS had a negative correlation, suggesting impaired pathway activity at lower levels of consciousness. Elevated lactate was linked to the "Response to Endogenous Stimulus," reflecting its role in metabolic stress. These results highlight the complex interplay between clinical features and biological pathways in DKA, with immune responses, metabolic regulation, and oxygen-related pathways playing significant roles.

## Discussion

In this study, we analyzed the plasma proteome of pediatric patients with severe DKA compared to age- and sex-matched insulin-controlled. After correcting for multiple comparisons, we identified 214 differentially expressed proteins with 162 upregulated and 52 downregulated (adj P < 0.05 and a fold change > 2). Using NLP, we mapped the expression patterns of these proteins across various organs (e.g., digestive, endocrine, neurological) and cell types (e.g., leukocytes, epithelial, endothelial). Pathway analysis revealed 38 enriched pathways, which were correlated with clinical variables, biochemistry, and GCS. This is the first comprehensive proteome profiling of pediatric DKA.

Our study specifically targeted patients with severe DKA (Segerer et al. 2021). The mean HbA1C level among these patients was 11.9%, indicating poor glucose control over the 2–3 months preceding the DKA episode (American Diabetes Association 2021). Additionally, these patients exhibited low HCO_3_ levels, characteristic of metabolic acidosis, along with elevated BUN, likely resulting from dehydration and catabolic processes.

Our analysis of the DKA plasma proteome identified a greater number of DEPs than previous studies focusing on specific inflammatory markers (Karavanaki et al. 2011; Omatsu et al. 2014; Woo et al. 2016a; Woo et al. 2016b). Consistent with our previous DKA research, we found highly elevated levels of PR3 (PRTN3; Woo et al. 2016b) and significant increases in MMP8 and MMP9 levels (Woo et al. 2016a). Many of the DEPs were linked to inflammation and metabolism. For example, interleukin-6 (IL6; upregulated) is a potent inducer of the acute phase response, while growth differentiation factor 15 (GDF15; upregulated) regulates food intake, energy expenditure, and body weight in response to metabolic and toxin-induced stresses, affecting cellular stress and β-cell function (Xu et al. 2022; Mohammad et al. 2023). Additionally, we noted a decrease in members of the TNF superfamily, some of which have been previously implicated in DKA (Rochfort et al. 2016).

The clinical manifestations of DKA are closely tied to organ-specific protein expression patterns. While early signs of organ dysfunction may not be immediately apparent, rapid deterioration can occur without prompt treatment. Common initial symptoms include polyuria, polydipsia, and polyphagia (Dhatariya et al. 2020; Alois and Rizzolo 2017). As DKA progresses, worsening ketosis and acidosis trigger systemic responses across multiple organs. Gastrointestinal symptoms such as nausea, vomiting, abdominal pain, and tenderness often arise, sometimes leading to upper gastrointestinal bleeding (Umpierrez and Freire 2002). Respiratory complications, including Kussmaul breathing, are common due to ketoacidosis and may, in rare cases, lead to spontaneous pneumothorax (Vallabhajosyula et al. 2016). Although less frequent, acute respiratory distress syndrome (ARDS) and pneumomediastinum, often associated with a cytokine storm, are serious complications (Horiya et al. 2022; Bialo et al. 2015).

Free thyroid hormone levels decrease as DKA severity increases, with acidosis contributing to this decline (Xing et al. 2021). The pituitary gland plays a key role in DKA pathogenesis, particularly in glucose metabolism and stress responses (Voss et al. 2019). Counter-regulatory hormones drive the metabolic disturbances that perpetuate the ketoacidosis cycle (Dhatariya et al. 2020; Voss et al. 2019). Our pathway analysis revealed significant enrichment in pathways related to "response to hormone" and "response to endogenous stimulus," indicating cellular and systemic changes in response to hormonal signals. Upregulated pathways involving peptide hormones and nitrogen-containing compounds suggest increased activity of the renin–angiotensin–aldosterone system and elevated levels of glucagon, catecholamines, cortisol, and corticotropin-releasing hormone during DKA (Voss et al. 2019). These hormones promote lipolysis, glycogenolysis, gluconeogenesis, and proteolysis, leading to the accumulation of nitrogen compounds. Combined with hyperglycemia and glycosuria, this contributes to dehydration and hyperosmolarity (Glaser 2005; Svart et al. 2016).

Lipid metabolism is significantly disrupted in DKA (Voss et al. 2019). Our results show marked changes in adipose tissue signaling, with increased signals for food intake and lipid mobilization into adipocytes, supported by elevated markers like GDF15, FABP4, FABP5, LDLR, and ANGPTL4 (Svart et al. 2016; Wang et al. 2021). Pathways related to lipid and fatty acid metabolism, particularly "lipid localization," "lipid transport," and "fatty acid transport," were significantly enriched. These findings align with known DKA pathophysiology, where counter-regulatory hormones drive lipolysis, mobilizing free fatty acids to the liver and worsening ketosis (Alois and Rizzolo 2017; Laffel 1999). Additionally, lipid mobilization and signaling may influence DKA-associated inflammation (Glass and Olefsky 2012).

DKA triggers systemic inflammation through the activation and recruitment of various leukocyte subtypes, even without infection. The severity of DKA is correlated with elevated levels of WBCs, neutrophils, and monocyte (Nichols et al. 2022). Our study confirms this association, showing leukocytosis in all DKA patients. We identified over thirty proteins linked to altered regulatory activities critical for inflammation, particularly those associated with leukocytes. Specifically, we observed increased levels of pro-inflammatory cytokines (e.g., IL6, IL15), innate immune components (e.g., PTX3, AZU1, CSF3), and markers of adaptive immune cells (e.g., IL1RL1, LEPR). In contrast, inhibitory hematopoietic mechanisms (e.g., RGMA, SMAD5, AREG) and anti-inflammatory pathways (e.g., CLEC4C, OGN) were downregulated. Pathways such as "signaling receptor regulator activity" and "signaling receptor activator activity" were significantly altered, reflecting enhanced pro-inflammatory signaling, leukocyte recruitment, and cytokine production. Enrichment in pathways related to "response to peptide hormone" and "signaling receptor activator activity" suggests increased extracellular and intracellular signaling. This immunological dysregulation can contribute to endothelial activation and increased vascular permeability, potentially leading to complications such as vasogenic edema (Omatsu et al. 2014; Woo et al. 2016b; Rochfort et al. 2016), rhabdomyolysis (Bialo et al. 2015), and acute pancreatitis due to repeated inflammation (Hahn et al. 2010).

In severe DKA, acute neurological dysfunction often manifests as a decreased GCS score, which can be worsened by cerebral edema (Eisenhut 2018; Bialo et al. 2015). Long-term neurological sequelae, such as neuronal damage and memory impairment, are linked to the severity of acidosis and are more pronounced in younger individuals (Bialo et al. 2015; Ghetti et al. 2020). Prolonged hyperglycemia further damages vascular structures, leading to cellular impairment and an increased risk of cerebrovascular events (Glaser 2005; Hamed et al. 2017). Our analysis revealed upregulation of pro-angiogenic factors (e.g., ADAMTS8) and pro-survival pathways (e.g., KITLG), alongside downregulation of pro-apoptotic factors (e.g., TNFSF12, TNFSF10), likely exacerbating inflammatory and vascular complications. We also observed increased levels of coagulation factors (e.g., HGF), platelet aggregation markers (e.g., TIMP4, SELP), and changes in cell-to-cell and cell-to-matrix interactions (e.g., ADAM23, SELP). Elevated levels of serine proteases (e.g., PR3, or PRTN3) and vasodilators (e.g., ADM, CALCA) further contribute to vascular derangements (Woo et al. 2016b; Hoffman et al. 2019).

Our analysis revealed both positive and negative associations between enriched pathways and clinical/biochemical variables. Elevated levels of HCO_3_ and GCS were negatively associated with several pathways. Specifically, HCO_3_ negatively correlated with pathways related to hormone response, receptor regulation of endogenous stimuli, and the metabolism of nitrogen, oxygen, and organonitrogen compounds, with weaker correlations in lipid metabolism. This is likely due to HCO_3_'s role in maintaining acid–base balance (Kamel and Halperin 2015). Metabolic acidosis may increase counterregulatory hormones like cortisol, glucagon, growth hormone, and catecholamines to elevate blood pH (Voss et al. 2019), while oxygen compounds support cellular respiration under hypoxia. Hyperventilation compensates for acidemia, and acidosis treatments can activate nitrogen pathways, potentially worsening the condition (Guh et al. 1997). GCS was negatively correlated with pathways linked to activity modulation, likely reflecting prolonged diabetes decompensation and its impact on signaling pathways and clinical outcomes (Dhatariya et al. 2020; Glaser 2005).

Positive correlations were most pronounced with lactate, WBC counts, and PO_2_. Lactate levels strongly correlated with receptor-ligand modulation, likely due to its role in regulating metabolic processes, such as insulin-mediated anti-lipolysis (Ahmed et al. 2010; Lu et al. 2011). Elevated lactate can result from anaerobic glycolysis in poorly perfused tissues, reduced hepatic clearance, stress-induced aerobic glycolysis, and mitochondrial dysfunction, all of which enhance cellular signaling (Lu et al. 2011; Liu et al. 2021). Elevated WBC counts likely reflect systemic inflammation, with no underlying infections identified (Aon et al. 2024; Hamtzany et al. 2023). Increases in specific subtypes, such as lymphocytes, may indicate enhanced cellular trafficking associated with metabolic inflammation (Dhatariya et al. 2020; Kamel and Halperin 2015). Activated lymphocytes promote inflammation and produce reactive oxygen species (Kitabchi et al. 2004). Additionally, changes in oxygen availability during metabolic acidosis may explain the positive correlation with PO_2_ (Kraut and Madias 2010).

Our study provides the first comprehensive evaluation of the DKA proteome, though several limitations must be addressed. First, while we included a balanced yet limited number of matched participants, our findings remain statistically significant after correction for multiple comparisons. Second, we focused solely on severe DKA patients to identify metabolic and inflammatory changes, but future studies should include a broader spectrum of DKA severity to improve generalizability. Third, although we identified differentially expressed proteins in plasma prior to insulin therapy, the lack of longitudinal samples prevents tracking changes in protein levels over time. This limits our ability to assess fluctuations during DKA treatment. Fourth, pediatric DKA patients experience osmotic diuresis, which could concentrate plasma proteins; this factor was considered in our analysis. Fifth, differences in T1D duration between cohorts, with some participants presenting with DKA as their first manifestation, could have influenced results. Lastly, despite significant associations between GCS and differentially expressed proteins, the limited GCS range warrants cautious interpretation of the data.

The identification of DEPs in severe DKA could improve patient stratification for future studies and clinical care. In this study, we identified 214 proteins and 38 signaling pathways that distinguish DKA patients from insulin-controlled diabetes. These findings provide valuable insights into the DKA pathophysiology. Future research should include longitudinal data to explore how initial protein profiles relate to short- and long-term outcomes.

## Supplementary Information

Supplementary Material 1.

## Data Availability

The datasets generated and/or analyzed during the current study are available from the corresponding author on reasonable request.

## Abbreviations

T1D: Type 1 diabetes
DKA: Diabetic ketoacidosis
CON: Control
IL: Interleukin
MMP: Matrix metalloproteinase
PR3: Proteinase-3
PEA: Proximity extension assay
NPX: Normalized protein expression
BMI: Body mass index
HbA1c: Hemoglobin A1C
BUN: Blood urea nitrogen
GCS: Glasgow coma scale
QC: Quality control
PCA: Principal component analysis
NLP: Natural language processing
DEPs: Differentially expressed proteins
FDR: False discovery rates
GO: Gene ontology
ORA: Over-representation analysis
FC: Fold change
OR: Odds ratio
WBC: White blood cell
