# Supplementary material for: Differential expression of plasma proteins and pathway enrichments in pediatric diabetic ketoacidosis
Source: Mol Med. 2025 Jan 7;31:4. doi: 10.1186/s10020-024-01056-7 (PMC11707870; doi:10.1186/s10020-024-01056-7)

Supplemental Table 1: GO Terms: DKA patients versus Insulin-controlled participants.

Id	Description	Ontology	Definition	Significant features	S	N	S/N (%)	OR	Z-score	P	P (adj.)
GO:0009725	response to hormone	BP	Any process that results in a change in state or activity of a cell or an organism (in terms of movement, secretion, enzyme production, gene expression, etc.) as a result of a hormone stimulus.	IL6; GDF15; AREG; ADM; COL1A1; MSTN; TIMP4; LEPR; REN; CRH; TNFSF10; IL1RN; TIMP3; TNFRSF11B; PCSK9; AGRP; ANXA3; IGFBP2; CCL21; PLA2G1B; FGF21; TSHB; HCLS1; TIMP1; EREG; CGA; KRT19; PKLR; PXN; IL10; REG1B; SPP1; SPINK1; NOS1; STAT5B; GAL; STC1; CA2; HSPA1A; REG1A; TFF1; IGFBP1; GCG	43	135	31.85185	2.999094	2.495923	2.29E-07	5.66E-04
GO:0009719	response to endogenous stimulus	BP	Any process that results in a change in state or activity of a cell or an organism (in terms of movement, secretion, enzyme production, gene expression, etc.) as a result of a stimulus arising within the organism.	IL6; GDF15; AREG; ADM; COL1A1; MSTN; TIMP4; LEPR; REN; CRH; TNFSF10; IL1RN; TIMP3; TNFRSF11B; PCSK9; AGRP; ANXA3; IGFBP2; CCL21; PLA2G1B; FGF21; TSHB; HCLS1; TIMP1; EREG; CGA; KRT19; PKLR; PXN; IL10; REG1B; SPP1; SPINK1; NOS1; STAT5B; GAL; STC1; CA2; HSPA1A; REG1A; TFF1; IGFBP1; GCG; VWC2; FOLR2; SMAD5; HGF; CALCA; RGMA; LDLR; ACTA2; IL12B; GPC1; CXCL8; SMOC2; CHRDL2; LPL; COMP; FURIN; NPPC; FSTL3; TGM2; KLB; APLP1; FST; AKT1S1; SHMT1; FCGR2B; CES1	69	273	25.27473	2.300953	1.75516	8.29E-07	0.001023
GO:0030545	signaling receptor regulator activity	MF	Binds to and modulates the activity of a receptor.	IL6; GDF15; AREG; ADM; MSTN; CRH; TNFSF10; IL1RN; TNFRSF11B; PCSK9; AGRP; CCL21; FGF21; TSHB; TIMP1; EREG; CGA; IL10; SPP1; GAL; STC1; HSPA1A; REG1A; TFF1; GCG; HGF; CALCA; IL12B; CXCL8; NPPC; FST; TNFSF11; KITLG; OSM; TNFSF12; CCN3; IL15; OGN; CSF3; TAFA5; CCL23; FLT3LG; LTA; PROK1; CCL20; HDGF; CCL24; IL18; OXT; CCL7; LIF; MIF; DKK4	53	201	26.36816	2.31637	1.481226	6.60E-06	0.004076
GO:0010876	lipid localization	BP	Any process in which a lipid is transported to, or maintained in, a specific location.	IL6; REN; CRH; PCSK9; PLA2G1B; SPP1; STAT5B; GAL; LDLR; LPL; FURIN; CES1; TNFSF11; MIF; FABP4; FABP5; MSR1; FABP1; RBP2; PLA2G2A; RBP5; FABP2; FABP6	23	60	38.33333	3.746262	1.936492	6.89E-06	0.004076

GO:0043434	response to peptide hormone	BP	Any process that results in a change in state or activity of a cell or an organism (in terms of movement, secretion, enzyme production, gene expression, etc.) as a result of a peptide hormone stimulus. A peptide hormone is any of a class of peptides that are secreted into the blood stream and have endocrine functions in living animals.	GDF15; AREG; ADM; COL1A1; MSTN; TNFSF10; PCSK9; AGRP; PLA2G1B; FGF21; TIMP1; EREG; PKLR; PXN; IL10; REG1B; SPINK1; STAT5B; GAL; CA2; REG1A; TFF1; IGFBP1; GCG	24	65	36.92308	3.534107	1.984556	9.22E-06	0.004076
GO:0048018	receptor ligand activity	MF	The activity of a gene product that interacts with a receptor to effect a change in the activity of the receptor. Ligands may be produced by the same, or different, cell that expresses the receptor. Ligands may diffuse extracellularly from their point of origin to the receiving cell, or remain attached to an adjacent cell surface (e.g. Notch ligands).	IL6; GDF15; AREG; ADM; MSTN; CRH; TNFSF10; IL1RN; TNFRSF11B; AGRP; CCL21; FGF21; TSHB; TIMP1; EREG; CGA; IL10; SPP1; GAL; STC1; HSPA1A; REG1A; TFF1; GCG; HGF; CALCA; IL12B; CXCL8; NPPC; TNFSF11; KITLG; OSM; TNFSF12; CCN3; IL15; OGN; CSF3; TAFA5; CCL23; FLT3LG; LTA; PROK1; CCL20; HDGF; CCL24; IL18; OXT; CCL7; LIF; MIF	50	189	26.45503	2.303499	1.309307	1.19E-05	0.004076
GO:0010033	response to organic substance	BP	Any process that results in a change in state or activity of a cell or an organism (in terms of movement, secretion, enzyme production, gene expression, etc.) as a result of an organic substance stimulus.	IL6; GDF15; AREG; ADM; COL1A1; MSTN; TIMP4; LEPR; REN; CRH; TNFSF10; IL1RN; TIMP3; TNFRSF11B; PCSK9; AGRP; ANXA3; IGFBP2; CCL21; PLA2G1B; FGF21; TSHB; HCLS1; TIMP1; EREG; CGA; KRT19; PKLR; PXN; IL10; REG1B; SPP1; SPINK1; NOS1; STAT5B; GAL; STC1; CA2; HSPA1A; REG1A; TFF1; IGFBP1; GCG; FOLR2; SMAD5; CALCA; LDLR; IL12B; CXCL8; LPL; COMP; NPPC; TGM2; APLP1; FST; SHMT1; FCGR2B; CES1; TNFSF11; KITLG; OSM; IL15; CSF3; CCL23; LTA; CCL20; CCL24; IL18; CCL7; MIF; FABP4; MSR1; IL1RL1; SELP; ADAM23; MNDA; MPO; BAG3; MMP8; LSP1; NADK; SERPINE1; CHI3L1; COL6A3; MMP9; ARG1; S100P; DNAJB1; IFNGR2; ALDH1A1; NPM1; PFKFB2	92	425	21.64706	1.943221	2.522371	1.29E-05	0.004076

GO:0030546	signaling receptor activator activity	MF	The function of interacting (directly or indirectly) with receptors such that the proportion of receptors in the active form is increased.	IL6; GDF15; AREG; ADM; MSTN; CRH; TNFSF10; IL1RN; TNFRSF11B; AGRP; CCL21; FGF21; TSHB; TIMP1; EREG; CGA; IL10; SPP1; GAL; STC1; HSPA1A; REG1A; TFF1; GCG; HGF; CALCA; IL12B; CXCL8; NPPC; TNFSF11; KITLG; OSM; TNFSF12; CCN3; IL15; OGN; CSF3; TAFA5; CCL23; FLT3LG; LTA; PROK1; CCL20; HDGF; CCL24; IL18; OXT; CCL7; LIF; MIF	50	191	26.17801	2.26642	1.302434	1.65E-05	0.004076
GO:0010243	response to organonitrogen compound	BP	Any process that results in a change in state or activity of a cell or an organism (in terms of movement, secretion, enzyme production, gene expression, etc.) as a result of an organonitrogen stimulus. An organonitrogen compound is formally a compound containing at least one carbon-nitrogen bond.	IL6; GDF15; AREG; ADM; COL1A1; MSTN; REN; CRH; TNFSF10; PCSK9; AGRP; PLA2G1B; FGF21; TIMP1; EREG; PKLR; PXN; IL10; REG1B; SPINK1; STAT5B; GAL; STC1; CA2; REG1A; TFF1; IGFBP1; GCG; FOLR2; LDLR; LPL; NPPC; TGM2; APLP1; SHMT1; FCGR2B; CES1; MMP9	38	131	29.00763	2.543726	1.747408	1.74E-05	0.004076
GO:0015849	organic acid transport	BP	The directed movement of organic acids, any acidic compound containing carbon in covalent linkage, into, out of or within a cell, or between cells, by means of some agent such as a transporter or pore.	PLA2G1B; FOLR2; CES1; TNFSF11; MIF; FABP4; FABP5; FABP1; RBP2; PLA2G2A; RBP5; FABP2; FABP6; ACE2; FOLR3	15	32	46.875	5.190576	1.59099	1.82E-05	0.004076
GO:0046942	carboxylic acid transport	BP	The directed movement of carboxylic acids into, out of or within a cell, or between cells, by means of some agent such as a transporter or pore. Carboxylic acids are organic acids containing one or more carboxyl (COOH) groups or anions (COO-).	PLA2G1B; FOLR2; CES1; TNFSF11; MIF; FABP4; FABP5; FABP1; RBP2; PLA2G2A; RBP5; FABP2; FABP6; ACE2; FOLR3	15	32	46.875	5.190576	1.59099	1.82E-05	0.004076
GO:0015908	fatty acid transport	BP	The directed movement of fatty acids into, out of or within a cell, or between cells, by means of some agent such as a transporter or pore. Fatty acids	PLA2G1B; TNFSF11; MIF; FABP4; FABP5; FABP1; RBP2; PLA2G2A; RBP5; FABP2; FABP6	11	19	57.89474	7.98875	2.064742	2.03E-05	0.004175

			are aliphatic monocarboxylic acids liberated from naturally occurring fats and oils by hydrolysis.							
GO:1901698	response to nitrogen compound	BP	NA	IL6; GDF15; AREG; ADM; COL1A1; MSTN; REN; CRH; TNFSF10; PCSK9; AGRP; PLA2G1B; FGF21; TIMP1; EREG; PKLR; PXN; IL10; REG1B; SPINK1; STAT5B; GAL; STC1; CA2; REG1A; TFF1; IGFBP1; GCG; FOLR2; LDLR; LPL; NPPC; TGM2; APLP1; SHMT1; FCGR2B; CES1; MMP9; NPM1; EGLN1	40	144	27.77778	2.397661	1.833333	3.20E-05	0.00605
GO:0015718	monocarboxylic acid transport	BP	The directed movement of monocarboxylic acids into, out of or within a cell, or between cells, by means of some agent such as a transporter or pore.	PLA2G1B; CES1; TNFSF11; MIF; FABP4; FABP5; FABP1; RBP2; PLA2G2A; RBP5; FABP2; FABP6	12	23	52.17391	6.353586	2.085144	3.43E-05	0.00605
GO:0006869	lipid transport	BP	The directed movement of lipids into, out of or within a cell, or between cells, by means of some agent such as a transporter or pore. Lipids are compounds soluble in an organic solvent but not, or sparingly, in an aqueous solvent.	REN; CRH; PCSK9; PLA2G1B; SPP1; GAL; LDLR; FURIN; CES1; TNFSF11; MIF; FABP4; FABP5; MSR1; FABP1; RBP2; PLA2G2A; RBP5; FABP2; FABP6	20	54	37.03704	3.498614	1.905159	5.20E-05	0.008141
GO:0098772	molecular function regulator activity	MF	A molecular function regulator regulates the activity of its target via non-covalent binding that does not result in covalent modification to the target. Examples of molecular function regulators include regulatory subunits of multimeric enzymes and channels. Mechanisms of regulation include allosteric changes in the target and competitive inhibition.	IL6; GDF15; AREG; ADM; MSTN; TIMP4; CRH; TNFSF10; IL1RN; TIMP3; TNFRSF11B; PCSK9; AGRP; ANXA3; CCL21; FGF21; TSHB; TIMP1; EREG; CGA; IL10; SPP1; SPINK1; NOS1; GAL; STC1; HSPA1A; REG1A; TFF1; GCG; HGF; CALCA; IL12B; CXCL8; FURIN; NPPC; FST; AKT1S1; TNFSF11; KITLG; OSM; TNFSF12; CCN3; IL15; OGN; CSF3; TAFA5; CCL23; FLT3LG; LTA; PROK1; CCL20; HDGF; CCL24; IL18; OXT; CCL7; LIF; MIF; DKK4; BAG3; SERPINE1; COL6A3; DNAJB1; ALDH1A1; NPM1; ANGPTL4; NCF2; SERPINA11; CST7; SERPINA9; DFFA; FETUB; GLRX; PEBP1; PPME1	76	343	22.15743	1.903953	1.835827	5.28E-05	0.008141

GO:1901700	response to oxygen-containing compound	BP	NA	IL6; GDF15; AREG; ADM; COL1A1; MSTN; REN; CRH; TNFSF10; PCSK9; AGRP; IGFBP2; CCL21; PLA2G1B; FGF21; TIMP1; EREG; PKLR; PXN; IL10; REG1B; SPP1; SPINK1; NOS1; STAT5B; GAL; STC1; CA2; REG1A; TFF1; IGFBP1; GCG; FOLR2; HGF; LDLR; IL12B; CXCL8; LPL; NPPC; TGM2; APLP1; SHMT1; FCGR2B; CES1; CSF3; LTA; CCL7; MIF; FABP1; SELP; MPO; MMP8; NADK; SERPINE1; COL6A3; MMP9; ALDH1A1; PFKFB2; EGLN1; GPR37	60	257	23.3463	1.962551	2.120862	9.49E-05	0.013763
GO:0015711	organic anion transport	BP	The directed movement of organic anions into, out of or within a cell, or between cells, by means of some agent such as a transporter or pore. Organic anions are atoms or small molecules with a negative charge which contain carbon in covalent linkage.	PLA2G1B; FOLR2; CES1; TNFSF11; MIF; FABP4; FABP5; FABP1; RBP2; PLA2G2A; RBP5; FABP2; FABP6; ACE2; FOLR3	15	36	41.66667	4.187318	1.5	1.00E-04	0.013763
GO:1901652	response to peptide	BP	NA	GDF15; AREG; ADM; COL1A1; MSTN; TNFSF10; PCSK9; AGRP; PLA2G1B; FGF21; TIMP1; EREG; PKLR; PXN; IL10; REG1B; SPINK1; STAT5B; GAL; CA2; REG1A; TFF1; IGFBP1; GCG; FCGR2B; MMP9	26	83	31.3253	2.744239	1.975757	1.09E-04	0.014177
GO:0015909	long-chain fatty acid transport	BP	The directed movement of a long-chain fatty acid into, out of or within a cell, or between cells, by means of some agent such as a transporter or pore. A long-chain fatty acid has an aliphatic tail containing 13 to 22 carbons.	PLA2G1B; MIF; FABP4; FABP5; FABP1; PLA2G2A; FABP2	7	10	70	13.34804	2.213594	1.41E-04	0.017429
GO:0033293	monocarboxylic acid binding	MF	Binding to a monocarboxylic acid, any organic acid containing one carboxyl (COOH) group or anion (COO-).	PLA2G1B; FABP4; FABP5; FABP1; RBP2; RBP5; FABP2; FABP6	8	13	61.53846	9.182266	2.218801	1.69E-04	0.01986
GO:0031406	carboxylic acid binding	MF	Binding to a carboxylic acid, an organic acid containing one or	PLA2G1B; NOS1; FOLR2; FURIN; SHMT1; FABP4; FABP5; FABP1; RBP2;	15	38	39.47368	3.816548	1.784436	2.09E-04	0.023448

			more carboxyl (COOH) groups or anions (COO-).	RBP5; FABP2; FABP6; SELP; FOLR3; EGLN1							
GO:0034774	secretory granule lumen	CC	The volume enclosed by the membrane of a secretory granule.	TIMP3; TIMP1; GCG; HGF; MIF; FABP5; MNDA; MPO; MMP8; SERPINE1; CHI3L1; ARG1; S100P; FOLR3; PTX3; AZU1; PRTN3; SRP14; S100A12; GUSB; VWF; PRSS2; RNASE3; CPPED1	24	77	31.16883	2.704873	2.507133	2.24E-04	0.024081
GO:0043177	organic acid binding	MF	Binding to an organic acid, any acidic compound containing carbon in covalent linkage.	PLA2G1B; NOS1; FOLR2; FURIN; SHMT1; FABP4; FABP5; FABP1; RBP2; RBP5; FABP2; FABP6; SELP; FOLR3; EGLN1	15	39	38.46154	3.654337	1.76141	2.94E-04	0.030238
GO:0005504	fatty acid binding	MF	Binding to a fatty acid, an aliphatic monocarboxylic acids liberated from naturally occurring fats and oils by hydrolysis.	FABP4; FABP5; FABP1; RBP2; RBP5; FABP2; FABP6	7	11	63.63636	10.00245	2.110579	3.38E-04	0.032864
GO:0060205	cytoplasmic vesicle lumen	CC	The volume enclosed by a cytoplasmic vesicle.	TIMP3; TIMP1; GCG; HGF; MIF; FABP5; MNDA; MPO; MMP8; SERPINE1; CHI3L1; ARG1; S100P; FOLR3; PTX3; AZU1; PRTN3; SRP14; S100A12; GUSB; VWF; PRSS2; RNASE3; CPPED1	24	79	30.37975	2.601847	2.475193	3.48E-04	0.032864
GO:0050729	positive regulation of inflammatory response	BP	Any process that activates or increases the frequency, rate or extent of the inflammatory response.	IL6; STAT5B; LDLR; IL12B; LPL; TNFSF11; OSM; IL15; LTA; CCL24; IL18; FABP4; PLA2G2A; IL1RL1; MMP8; SERPINE1; S100A12	17	48	35.41667	3.219654	1.299038	3.69E-04	0.032864
GO:0009914	hormone transport	BP	The directed movement of hormones into, out of or within a cell, or between cells, by means of some agent such as a transporter or pore.	IL6; REN; CRH; IL1RN; CGA; SPP1; SPINK1; GAL; GCG; TNFSF11; OSM; CCN3; LIF; NADK; PFKFB2; PSMD9	16	44	36.36364	3.34652	1.206045	3.92E-04	0.032864
GO:0046879	hormone secretion	BP	The regulated release of hormones, substances with a specific regulatory effect on a particular organ or group of cells.	IL6; REN; CRH; IL1RN; CGA; SPP1; SPINK1; GAL; GCG; TNFSF11; OSM; CCN3; LIF; NADK; PFKFB2; PSMD9	16	44	36.36364	3.34652	1.206045	3.92E-04	0.032864
GO:0023061	signal release	BP	The process in which a signal is secreted or discharged into the extracellular medium from a cellular source.	IL6; REN; CRH; IL1RN; CGA; SPP1; SPINK1; GAL; GCG; TGM2; TNFSF11; OSM; CCN3; LIF; MIF; NADK; PFKFB2; PSMD9; SNCG	19	57	33.33333	2.947917	1.456986	4.07E-04	0.032864
GO:0046883	regulation of hormone secretion	BP	Any process that modulates the frequency, rate or extent of the	IL6; REN; CRH; SPP1; SPINK1; GAL; GCG; TNFSF11; OSM; CCN3; LIF; NADK; PFKFB2; PSMD9	14	36	38.88889	3.708353	1	0.000413	0.032864

			regulated release of a hormone from a cell.								
GO:0031983	vesicle lumen	CC	The volume enclosed by the membrane or protein that forms a vesicle.	TIMP3; TIMP1; GCG; HGF; MIF; FABP5; MNDA; MPO; MMP8; SERPINE1; CHI3L1; ARG1; S100P; FOLR3; PTX3; AZU1; PRTN3; SRP14; S100A12; GUSB; VWF; PRSS2; RNASE3; CPPED1	24	80	30	2.553094	2.459675	4.31E-04	0.033228
GO:0031329	regulation of cellular catabolic process	BP	Any process that modulates the frequency, rate or extent of the chemical reactions and pathways resulting in the breakdown of substances, carried out by individual cells.	IL6; TIMP4; LEPR; TIMP3; PCSK9; FGF21; TIMP1; IL10; HGF; RGMA; LDLR; FURIN; FABP1; BAG3; NPM1; DFFA; APEX1; NMNAT1; CARHSP1; SIRT2	20	62	32.25806	2.812266	2.032002	4.68E-04	0.035009
GO:0005125	cytokine activity	MF	The activity of a soluble extracellular gene product that interacts with a receptor to effect a change in the activity of the receptor to control the survival, growth, differentiation and effector function of tissues and cells.	IL6; GDF15; AREG; MSTN; TNFSF10; IL1RN; TNFRSF11B; CCL21; TIMP1; IL10; SPP1; IL12B; CXCL8; TNFSF11; KITLG; OSM; TNFSF12; IL15; CSF3; TAFA5; CCL23; FLT3LG; LTA; CCL20; CCL24; IL18; CCL7; LIF; MIF	29	106	27.35849	2.26181	0.874157	6.11E-04	0.043297
GO:1901701	cellular response to oxygen-containing compound	BP	NA	IL6; GDF15; COL1A1; MSTN; CRH; PCSK9; PLA2G1B; FGF21; PKLR; PXN; IL10; SPP1; SPINK1; STAT5B; STC1; CA2; IGFBP1; GCG; FOLR2; HGF; LDLR; IL12B; CXCL8; LPL; NPPC; TGM2; APLP1; SHMT1; FCGR2B; CES1; CSF3; CCL7; MIF; FABP1; MPO; MMP8; NADK; SERPINE1; MMP9; ALDH1A1; GPR37	41	168	24.40476	1.980686	1.928792	6.22E-04	0.043297
GO:0007631	feeding behavior	BP	Behavior associated with the intake of food.	GDF15; LEPR; REN; AGRP; GAL; GCG; CALCA; ACE2	8	15	53.33333	6.547502	1.549193	6.39E-04	0.043297
GO:0023051	regulation of signaling	BP	Any process that modulates the frequency, rate or extent of a signaling process.	IL6; GDF15; ADM; COL1A1; MSTN; REN; CRH; TNFSF10; IL1RN; PCSK9; IGFBP2; CCL21; PLA2G1B; FGF21; HCLS1; TIMP1; CGA; PXN; IL10; SPP1; SPINK1; NOS1; GAL; CA2; HSPA1A; IGFBP1; GCG; VWC2; SMAD5; HGF; RGMA; LDLR; ACTA2; IL12B; GPC1; CXCL8; SMOC2; CHRDL2; FURIN; NPPC; FSTL3; TGM2; KLB; APLP1; FST; AKT1S1; FCGR2B; TNFSF11; KITLG; OSM; TNFSF12; CCN3; CSF3; CCL23; PROK1;	92	464	19.82759	1.658444	1.949801	6.49E-04	0.043297

				CCL20; CCL24; IL18; CCL7; LIF; MIF; DKK4; FABP5; PLA2G2A; SELP; MMP8; NADK; SERPINE1; CHI3L1; MMP9; ARG1; NPM1; PFKFB2; ACE2; PEBP1; GPR37; S100A12; VWF; PSMD9; SNCG; NMNAT1; SIRT2; KAZALD1; DLK1; CDON; ROR1; PLAT; PIK3AP1; RASSF2; TNFRSF12A; CLEC4D; MYOC							
GO:0031330	negative regulation of cellular catabolic process	BP	Any process that stops, prevents, or reduces the frequency, rate or extent of the chemical reactions and pathways resulting in the breakdown of substances, carried out by individual cells.	TIMP4; LEPR; TIMP3; TIMP1; IL10; HGF; FURIN; DFFA; NMNAT1; SIRT2	10	22	45.45455	4.800995	2.132007	6.75E-04	0.043874

Individual GO terms were assessed by evaluating whether the number of significantly differentially expressed proteins within a term (**S**) was more than what would be expected by chance given the total number of proteins (**N**). A P-value (**P**) was determined using a hypergeometic test performed using the clusterProfiler package and adjusted for multiple comparisons using the Benjamini-Hochberg method (**P (adj.)**). Significance was considered as possessing an adjusted P-value < 0.05 and ≥1 features per set. An odds ratio (**OR**) was calculated as the number of significant proteins observed over that which could be expected by chance. A term activity Z-score was calculated as $Z=(Su-Sd)/\sqrt{N}$, where N is the total number of proteins in the term, and **Su** and **Sd** are the number of significant up-regulated and down-regulated proteins in the term, respectively.

SUPPLEMENTAL FIGURE 1: Correlations between the top 50 differentially expressed proteins and clinical variables.

Correlations between the top 50 differentially expressed proteins and clinical variables. The heatmap colour represents the value of the Pearson correlation coefficient, where a value of -1 represents a perfectly inverse correlation and a value of 1 represents a perfectly direct correlation. The stars (*) in the cells indicate that the Pearson correlation for the given pairing is significant at the $p < 0.01$ (***), $p < 0.05$ (**), or $p < 0.1$ (*) level. Note the number of significant correlations against white blood cell count (WBC), Lactate, and pO_2.

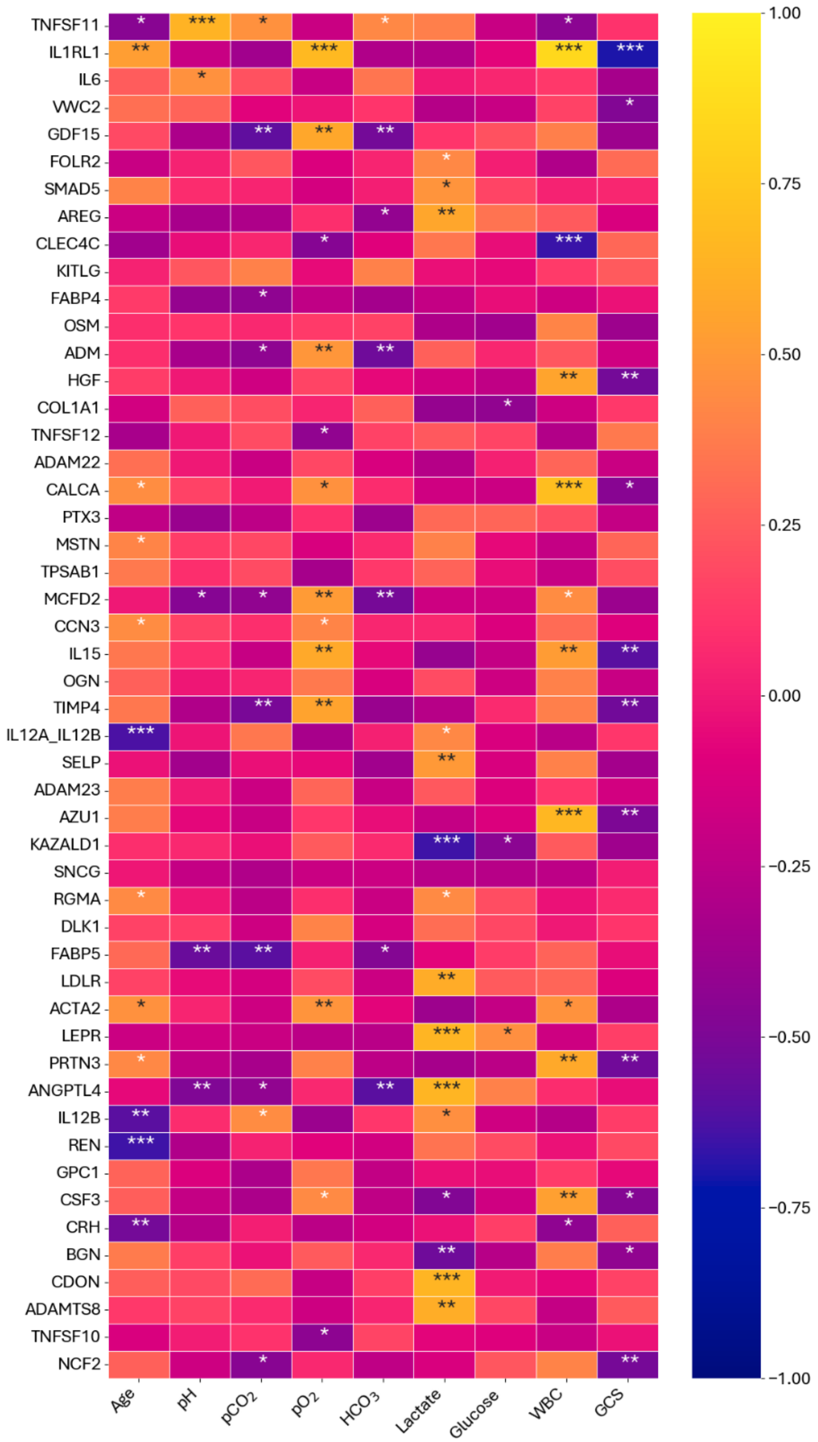

Supplement: Supplementary file 1 — Supplementary Material 1. [file 10020_2024_1056_MOESM1_ESM.pdf]
